# Sleep in Residential Aged Care: A Secondary Qualitative Analysis of Data from the Australian Royal Commission into Aged Care Quality and Safety

**DOI:** 10.1177/01939459251324831

**Published:** 2025-03-03

**Authors:** Aisling Smyth, Patricia Cain, Sabine Pangerl, Christopher Gordon, Kasia Bail, Davina Porock

**Affiliations:** 1 Centre for Research in Aged Care, School of Nursing & Midwifery, Edith Cowan University, Perth, WA, Australia; 2 School of Nursing & Midwifery, Faculty of Health, University of Canberra, Bruce, ACT, Australia; 3 Centre for Ageing Research and Translation, University of Canberra, Bruce, ACT, Australia; 4 Centre for Sleep and Chronobiology, Woolcock Institute of Medical Research, Macquarie University, Sydney, NSW, Australia

**Keywords:** sleep, residential aged care, nursing home, person-centered care, patient-centered care, long-term care

## Abstract

**Background:**

Sleep is a crucial healing and restorative component of older person care but is often negatively impacted through the effects of institutionalization in residential aged care (RAC). Currently, information about how sleep occurs is limited.

**Methods:**

Through the lens of person-centered care, this study examined submissions to the Australian Royal Commission into Aged Care Quality and Safety. The Commission was established in 2018 with the aim of protecting and improving the safety, quality of life, and well-being of people receiving aged care. The commission received 10 000+ submissions from a range of stakeholders, including consumers, family members, expert witnesses, healthcare professionals, and industry. Using a big qualitative data approach with 33 sleep-related keywords, submissions were analyzed using thematic analysis to understand how institutional practices impact individual sleep experiences.

**Results:**

Three themes were identified highlighting tensions between institutional requirements and person-centered care: (1) Care practices of RAC staff may impact residents’ sleep, (2) Tensions between provision of overnight care and preservation of sleep, and (3) The dignity of choice of residents including autonomy of sleep timing and their sleep environment.

**Conclusion:**

Opportunities to improve the quality of sleep experience and associated outcomes in Australian RAC exist. Improved staffing, planning for person-centered timing of care, and addressing the competing tensions of safety surveillance activities and person-centered care implementation in a home environment are needed.

Sleep has a fundamental role in promoting optimal health and well-being across the lifespan. Sleep is not a passive process but rather an active process of rest, repair, and rejuvenation. Sleep disturbances in older adults are commonplace, with up to 50% of people over 65 reporting suboptimal sleep quality and quantity.^1^ Older people in residential aged care (RAC, also known as nursing homes or long-term care facilities) experience worse sleep than their community-dwelling counterparts.^2^ Poor sleep can exacerbate many of the complex health issues already present in RAC residents such as cognitive decline,^3^ increased falls,^4^ increased frailty, higher mortality,^5^ and decreased psychological well-being.^6,7^ Sleep in RAC is known to be impeded by a variety of factors including environmental factors such as light or noise, sleep disruption due to other residents or staff, and decreased sleep drive due to excessive napping or decreased physical activity.^8^ Given the known association between poor sleep and health and well-being, the preservation and optimization of sleep for older adults are paramount. Moreover, the RAC workforce and stakeholders have a pivotal role in the assessment, management, and promotion of quality sleep. Despite sleep being fundamental to health and well-being, there is limited data characterizing sleep practices and experiences within RAC settings, particularly from a person-centered perspective.

This study is grounded in the person-centered care framework,^9^ a philosophy that positions the individual at the center of care delivery, promoting autonomy and active involvement in care decisions. Within RAC, this framework highlights the tension between institutional care requirements and individual wants and needs. Person-centered care principles suggest that sleep practices should be individualized rather than standardized while acknowledging the practical constraints of the residential care environment. This conceptual lens guided our analysis of how current sleep-related practices in RAC align with or deviate from person-centered ideals, providing invaluable insight into an understudied area.

The Australian Royal Commission into Aged Care Quality and Safety (henceforth Royal Commission) was established by the Australian Governor-General in 2018 following the exposure of serious failings in the aged care sector.^10^ The terms of reference for the Royal Commission were broadly to identify the quality of aged care services and identify areas of challenge and opportunity with a view to understand “how best to deliver aged care services.”^11^ Over 10 000 documents were submitted by the public, including aged care consumers, family members, carers, health professionals, and providers, and over 100 public hearings were held.^11^ In 2019 a 3-volume Interim Report was released and in 2021, an eight-volume final report was released; in total the Royal Commission made 148 recommendations and a call for fundamental reform to the Australian aged care system.^11^

There is limited data characterizing the sleep practices and sleep health of residents in RAC within Australia and beyond. However, the existing literature indicates that a high proportion of residents experience suboptimal sleep, further exacerbated by environmental disruption, altered circadian alignment, and uncontrolled pain.^12^ Given the paucity of current evidence exploring sleep assessment, promotion, and management in RAC, and the known prevalence of poor sleep in older adults within RAC, the Royal Commission data represented a unique opportunity to examine how institutional practices impact individual sleep experiences. While sleep was not a specific focus of the Commission’s recommendations, we anticipated that sleep-related concerns would emerge across submissions from key stakeholders, including healthcare professionals, residents, their families, and care providers, offering insight into how well current practices align with person-centered care principles.

## Purpose

In this study, we aimed to utilize the Royal Commission data set to identify and describe factors impacting residents’ sleep practices and experiences. Through the lens of person-centered care, we sought to understand how institutional routines and individual preferences intersect in relation to sleep, with a view to identifying opportunities for improvement in sleep-related care practices within RAC settings.

## Method

Evidence generated from the submissions and proceedings of the Royal Commission is currently available in the public domain.^11^ Despite having unrestricted access, due to the sensitive nature of the evidence and the consideration that this content was not intended for research purposes, we gained approval from the Royal Commission to use the publicly available content as data for scholarly analysis. The Edith Cowan University Human Research Ethics Committee also provided approval for the project.

For this analysis we included the documents most likely to feature content and discussion related to sleep; these were general submissions (1183 submissions from individuals and organizations), hearing transcripts, and post-hearing submissions (227 verbatim transcripts of hearings where witnesses were questioned), exhibits (5712 items including witness statements and supporting documents for hearings), and commissioner reports (17 reports including the interim and final reports). In total, we downloaded 11 GB of data to NVivo for storage and analysis. Undertaking qualitative inquiry on this volume of data was not practical. Rather than sifting through all documents for instances relating to sleep, we adopted strategies suited to big qualitative data and used keywords to curate a topic-specific dataset.^13^ Using the extant literature as a guide, a list of 33 keywords (with abbreviations and Boolean logic) and keyword combinations relating to sleep was developed. The list included common terms such as sleep*, nap*, *bedtime*, *rest*, and disturb* together with more clinical terms such as insomnia*, *sleep assessment*, and *light therapy*. Previous research documented the use of chemical restraints within the same dataset^14^ and therefore was omitted from this research. However, search terms specific to sleeping medications were included. A full list of the search terms can be found in the Supplemental File.

All keywords were systematically searched across general submissions, hearing transcripts, and commissioner reports with the number of mentions for each keyword recorded. The exhibits were searched last. Due to the volume of exhibits, we proceeded with “economy of effort,”^15^ restraining our search to the keywords that had returned the most mentions in the previous searches. The text identified from the keyword searches was reviewed and relevant content was captured to create a dedicated topic-specific dataset for analysis. In reviewing the content, all sources and voices were treated as equally important.

Descriptive thematic analysis of the topic-specific data set was undertaken and followed the six-step framework of Braun and Clarke.^16^ The data were read, reread, coded, and themed by one researcher (S.P.), with a second and third researcher reviewing and refining the themes (A.S., P.C.) until an agreement was reached. Themes were illustrated with the support of extracted quotes. We followed ethical guidelines, omitting names of individuals and organizations in our extract reporting, as per the Association of Internet Researchers’ standards.^17^ Sources of quotes are identified by document type in parentheses following the quote.

## Results

From the data analysis, 3 overarching themes were identified: (1) RAC staffing practices impact residents’ sleep, (2) Tension between provision of resident care and protection of overnight sleep, and (3) Challenges to person-centered sleep practices. Within each of the themes, we explore a total of 7 distinct subthemes to isolate the specific concerns expressed through the data. Each theme is supported with extracts from hearing transcripts, general submissions, post-hearing submissions, and exhibits. Extracts include the perspectives of residents, family members, stakeholders, and healthcare workers. Where extracts lack clarity or context, words have been inserted, indicated by square brackets.

### Care Practices of RAC Staff May Impact Resident Sleep

RAC staff practices can profoundly impact resident sleep, for the better or for the worse. The first theme identifies numerous staff practices that can directly impact sleep. These include effective nursing care planning, time-sensitive provision of care to protect natural sleep time, and staff utilizing the bed as a “restraint” mechanism for residents, thus impeding natural sleep routines.

#### Inconsistent sleep care planning

Sleep assessment and management are crucial aspects of care for older people in RACs. The first extract is an example of some of the reported positive practices, such as conducting sleep assessments on admission and on a regular basis, developing care plans that incorporate sleep needs and preferences, and evaluating care plans consistently.

Care recipients’ sleep patterns, including settling routines and personal preferences, are identified through assessment processes on entry. Care plans are developed and reviewed to ensure strategies to support natural sleep remain effective. (Site Audit Report, Exhibit)

However, informants to the Royal Commission identified areas of concern including the lack of re-assessment and evaluation of sleep care plan strategies, inadequate documentation of sleep disturbances, lack of strategies for managing sleep disturbance, and care plans that did not include individual sleep patterns and preferences.

Care recipients’ sleep patterns are not being identified and an appropriate care plan developed, and care recipients are not being assisted to achieve natural sleep patterns. (Site Audit Report, Exhibit)

Staff practice does not always support care recipients’ natural sleep patterns. Staff have not had any training to assist with respecting care recipient needs and preferences. (Site Audit Report, Exhibit)

It is evident from the data that sleep assessment and sleep management are not consistently routine or established features of care plans. Our findings are evidence that sleep assessments may be lacking from the outset, or they may not be maintained, indicating that adhering to formal policies and practices to promote quality sleep is not an industry-wide priority.

#### Staffing levels dictate timing of care provision

Several sources reported staff shortages during critical times of the day, such as when residents are being showered, getting up in the morning, and going to bed at night. There were concerns about the carer-resident ratio and a lack of registered nurses on duty during afternoon and night shifts resulted in a breakdown of communication and a lack of clinical care for high-care residents who required extensive support.

By the time you get to 5 o’clock the next morning in a nursing facility such as the one mum’s in, the communication has broken down to a point where the morning staff coming on at 5 am have no idea that she has been asleep since midday the previous day. (family member, hearing transcript)

Further, the carers’ perception was that they were coerced into completing specific tasks as a means of managing their workloads, which was regarded as a form of managerial harassment.

Management harassment and bullying to meet work demands such as waking residents for medication and showering before 6.30am to reduce workload of morning shift. (Australian Nursing and Midwifery report, hearing transcript)

These staff were expected to wake residents at 0500 to commence the personal hygiene tasks. If they didn’t do this, the morning PCAs would be openly angry because they didn’t have time and weren’t able to help all the residents with their personal hygiene according to their needs. Both morning and afternoon staff were rushed and, therefore, the residents were rushed (Australian Nursing and Midwifery report, hearing transcript)

The impact of inadequate staffing on residents’ sleep health is significant because it not only directly affects the well-being of residents but also can have far-reaching consequences for safety, emotional health, regulatory compliance, and overall quality of care within RAC.^18^

#### Impact of restrictive practices on residents’ sleep

Restrictive practices refer to any intervention that impedes a person’s freedom of movement or ability to make a decision.^19^ Our findings revealed evidence of residents being left in bed for extended periods of time during the day, tantamount to environmental restraint. This practice of excessive time in bed is counterproductive to supporting optimal sleep environments and routines for residents, with a basic tenet of sleep hygiene being the avoidance of excessive time in bed during the day or when not sleeping.

As the subsequent extracts demonstrate, those bearing witness considered low staffing levels and inadequate capacity to provide person-centered care as the reasons behind limiting residents to their beds for prolonged periods.

The breaking point came when at 2pm in the afternoon I found her still in bed, with her continence aid on the floor. She had not been showered or dressed for the day, and when I questioned the staff member, she stated that she had 17 residents to attend to. (Family member, general submission)

. . . residents were confined to bed for up to 12 hours due to understaffing . . . (Stakeholder, hearing transcript)

As well as environmental restraints, family members and care staff recounted witnessing the use of physical restraints and discussed how this related to residents’ sleep overnight. The following extracts illustrate how the use of physical restraints was associated with the inability of residents to establish and maintain natural sleep routines.

. . .they would start to restrain him at 3 o’clock in the morning to sit in a chair, and then told us that he had trouble sleeping. I would have trouble sleeping tied into an upright chair, of course. (Family member, hearing transcript)

. . . he was physically restrained for periods of between 30 mins and 2 hours at a time, in aggregate daily periods varying from several hours to 13 or 14 hours. He was generally restrained during the day because he was falling asleep and during the night because he was restless and wandering. (RAC Director, hearing transcript)

Care staff also reported their observations and concerns over residents’ sleep routines. Some of the reported events appear to show evidence of behavioral symptoms of dementia, which routinely impact sleep routines. As indicated by the next extracts, there appears to have been a minimal attempt to employ behavioral strategies to improve sleep routines; rather, physical restraint strategies were employed. This begs the question as to whether RAC staff have the knowledge and resources to identify and manage sleep behaviors associated with dementia/cognitive decline.

Sometimes he just refused to sleep and just continued to walk, and then next, at times night-time, he may be restrained. (Care worker, Hearing transcript)

He wandered at night unless diverted by nursing staff, then was drowsy during the day as a result of day-night reversal. The primary issue in this case study was the use of physical restraints. . . (RAC Director, Hearing transcript)

Here, we identified concerning care-restrictive practices in some RACs that may arise from poor resident sleep (physical restraints) and/or contribute to further negative impacts on residents’ quality of sleep (environmental restraints). The use of restrictive practices may negatively impact the ability of older people to be supported with positive sleep environments and sleep hygiene routines.

Aged care providers are faced with the challenge of adapting to the complex and continuously changing care needs of their residents, with ever-challenging staffing ratios. This theme reveals the potential role RAC staff can play in either impeding or optimizing the sleep of residents via sleep care planning, protection of sleep routines, and supporting natural sleep-wake rhythms. Furthermore, staffing levels and workload resulted in the timing of morning hygiene care being dictated by staffing capacity, rather than when residents were awake and/or wanted hygiene care support. Lastly, the use of physical and environmental restraints within RAC may arise from poor resident sleep and serve to further exacerbate these issues.

### Tensions Between Provision of Resident Care and Preservation of Sleep

The second theme highlights the tensions that exist between the need for appropriate care routines overnight and minimizing disturbance so that older people can enjoy a good night’s sleep. The 2 subthemes draw attention to balancing the challenges of mitigating risk and promoting safety, with not disturbing residents overnight.

#### Nocturnal monitoring at odds with quality sleep

As indicated by the first theme, there is an incongruity in how aged care providers are assessing and managing residents’ natural sleep patterns. In some cases, residents’ sleep preferences are documented in their care plan, with care staff offering support to help people settle in the evening, and sleep disturbances are investigated and remedied whenever possible. However, despite such efforts, our findings show that the provision of overnight care and monitoring may be overly disruptive and not consistent with residents’ preferences.

On some occasions, the staff come into my room at 11pm to check on me and after this I can’t go to sleep. This makes getting a good night’s sleep generally quite hard because staff always also enter my room to refill supplies in my bathroom in the early hours of the morning. (RAC resident, exhibit)

In the morning at 5 o’clock they come in as well, and to bring pads in and put them in the thing behind the door. That wakes you up but I’m usually awake at 5 o’clock so it’s not so bad. . . They knock on the door and walk straight in. They don’t wait for a response. (RAC resident, hearing transcript)

Furthermore, care staff identified that conducting observations during nocturnal hours may considerably undermine residents’ sleep quality and is incompatible with the principles of person-centered care.

We’re often waking them [residents] in the middle of the night to continue doing these observations, and I think this really impinges on quality of life, and it’s definitely not person-centered care. (Nursing staff, Hearing transcripts)

Don’t just shut the door, then disturb the resident every couple of hours depending on their care plan and then leave. Have a better way of keeping watch on the resident during the night e.g. CCTV (Family member, Submission)

I’m a very bad sleeper. . .sometimes we’ve had staff come into your room, and they open the door and look to see if you’re in bed. And I’ve said, “I get to bed on my own, nobody helps me. Why do you have to do that?” “We have to do that.” I say, “No, you don’t have to do it because I won’t get back to sleep for the rest of the night.” (RAC resident, hearing transcript)

Both care staff and residents recognize the impact of overnight care on quality sleep. Concerns are expressed here by both care recipients and care providers. Care workers are aware of the impact of nocturnal monitoring on sleep quality, recognizing this as at odds with the delivery of person-centered care.

#### Balancing care routines, repositioning, and rest

The routine practices of continence care and repositioning at night in RACs have been reported throughout the data as a significant disruption to the natural sleep patterns of residents. Progress notes for individual care recipients showed that sleep disturbance was a common issue due to repositioning at night and continence care and often resulted in care recipients being unable to settle again. Family members also highlighted the importance of minimizing the number of times care recipients are disturbed at night.

Disturbing the patient when they were finally comfortable and sleeping when perhaps feeding, medication and toileting could have happened once and not several times. (Family member, General submission)

To avoid nocturnal continence care disruption, some providers have taken practical measures, such as using continence pads with an 8-hour capacity, to facilitate longer undisturbed sleep. Further, the pragmatic and effective solution of a larger catheter bag was implemented, but only after a complaint from a family member:

They have now also agreed to give him a larger catheter bag so they only have to empty it in the morning. (Family member, exhibit)

Similarly, night-time repositioning to avoid pressure injuries can be a major disruptor of sleep patterns. This is another point of tension where constantly waking residents for repositioning can also mean high levels of disturbance leading to poor quality sleep and sleep deprivation. In one account, even the introduction of an alternating pressure air mattress designed to reduce the need for repositioning and minimizing sleep was not enough to facilitate less disruption, as indicated by the following statement from a family member.

. . . even though he is on an air-mattress at night, they insist on moving him every two hours. It wasn’t until I spent the night in his room. . .that I realized how much sleep deprivation he has. Staff would burst into his room, put the main light on, click off the foot brakes on his bed in order to pull it from the wall (which would make loud clacking noises), pull off his doona, grab him and roll him to one side to reposition him (which caused him to think he was being tipped off the bed). (Family member, exhibits)

This subtheme highlights the tension of balancing potential risk, by reducing continence care and repositioning frequency, with increasing opportunity for good quality and quantity of sleep by reducing the frequency of overnight care.

Overnight clinical care and sleep quality are the focus of this theme. The data included repeated concerns, from multiple perspectives, relating to how current care practices are not doing enough to support and improve sleep quality for residents. Overnight care is often not person-centered, and instead, there are arbitrary times at which residents are woken for “checks,” repositioning, and continence care. When taken together with the first theme which identified the provision of early morning personal care, this presents a picture of interrupted sleep overnight, through to early morning time.

### Challenges to Person-Centered Sleep Practices

The Aged Care Quality Standard 1 is consumer dignity and choice, requiring older people to be treated with respect and to be supported to make informed choices about individual care.^20^ This theme identified how shortfalls in providing respectful care contribute to negative impacts on residents’ sleep quality and also their sense of autonomy. Two subthemes are presented: (1) Too early to bed, too early to rise? and (2) Bright, crowded, noisy, and not person centered.

#### Too early to bed, too early to rise?

The Royal Commission data highlighted the ways in which older people in RAC are denied the dignity of being able to set their own rise times and bedtimes and are instead being routined to times more akin to the bedtimes of very young children. As the succeeding extracts demonstrate, some facilities have a practice of enforcing early and unnecessary bedtimes and rise times, effectively denying residents the autonomy to choose their preferred sleep routine timing.

All residents were put into bed in the evening by 7pm, without any choice or decision in relation to what they wanted. (Aged care worker, submission)

A younger man in a residential facility, who on occasion wants to have a sleep in but is made to get out of bed. Being non-verbal, his only means of expressing his displeasure with this decision is to resist physically and then he gets branded as “aggressive.” (COTA, exhibit)

Imposing arbitrary restrictions meant that residents were in bed at times when they were not tired and woken early in the morning when not ready, effectively interfering with residents’ natural sleep cues. The practice of enforcing early bedtimes also infantilizes older people.

I felt like I had no choice left in my life. There were set times for eating meals, waking up, and going to sleep. (RAC resident, exhibit)

Similar to the previous subthemes above, inadequate staffing levels and training may serve as explanations for the diminished capacity to deliver person-centered care. However, this practice may be reflective of the broader practice of infantilizing older people^21^ and reflects the lived experience of older people in residential care being unable to advocate for their personal needs, including sleep preferences, and may be reflective of staff indifference, negative attitudes, and ageism.^22^

#### Bright, crowded, noisy, and not person centered

The second element of this overarching theme focuses on the impact of the physical sleep environment in residential care and how the institutional nature of the sleep setting has a significant impact on the well-being of residents. Reports throughout the data indicated that the overall environment within many RACs was not conducive to sleep. Rather, as the following extracts demonstrate, issues were raised in relation to uncontrolled noise and lighting at night and during night-time resident care.

[Resident] told us that she had difficulty sleeping due to the light in the hall shining on her eyes. (exhibit)

[We received] information about the environment being noisy, including due to call bells. . .also. . .the older style beds make a noise when put up/down. (exhibit)

Informants to the Royal Commission also considered shared rooms to be disruptive to the sleep and overall health of those in the shared spaces.

There was a second bed in the room, but it was not always occupied. When it was, it was stressful to her because the other resident was known to scream all night, or otherwise cause problems. (Family member, general submission)

In addition, the data revealed that many residents have disrupted sleep/wake cycles, leading to daytime drowsiness as well as night-time wakefulness. In addition, there were frequent reports of disrupted sleep due to disturbances carried out by other residents with what appears to be sleep disruption associated with symptoms of dementia.

. . .it wasn’t a quiet room. It—you could still hear everything that was going on. There were often 5 residents screaming both day and night. I often closed the door if we could. (Family member, hearing transcript)

Sleep disturbances are a frequent occurrence among individuals with dementia or depression.^23,24^ In the examples presented here, the concerns are mostly expressed by those who have experienced a disturbance in their sleep. However, equally important to recognize here is that such disturbances have occurred due to the complexity of care and potentially a lack of person-centered care to support residents with cognitive decline, depression, or dementia.

## Discussion

This study conducted a secondary qualitative analysis of a large data set of evidence collected during the Australian Royal Commission. This work allowed the collation of data relating to sleep in Australian RAC services to provide new insights into sleep-related practices. The key themes identified centered around the role of RAC staff care practices, overnight care timing, and autonomy of choice relating to sleep for residents. Through our person-centered conceptual framework, the findings highlight how institutional imperatives often override individual sleep preferences and needs. The tension between standardized care protocols and person-centered principles manifests clearly in practices around sleep timing, overnight monitoring, and environmental controls.

Within this person-centered framework, optimization of sleep in RAC is of the utmost importance, as disrupted sleep in older adults increases the risk of frailty, mortality, and morbidity.^25^ However, the residential aged care setting is filled with barriers to good overnight sleep including overnight “checks,” continence care, repositioning of clients, limited physical activity, excessive napping time/time in bed, and environmental issues such as light, noise, and other residents.

While provision of overnight care is often necessary, previous research has identified that rather than a person-centered, individualized approach, overnight care such as continence care and repositioning is often performed at regular, arbitrary pre-defined intervals, thus limiting the opportunity for uninterrupted sleep periods for residents.^26-28^ Further examination of how to manage risks and benefits of potentially reducing overnight care interruptions for individual residents, how to promote recognition of the dignity of risk, and how to enable shared understanding and acceptance of risk due to decreased overnight care is needed to fully address these issues.^29^

Residents’ own preference for night-time routines, including settling times, wind-down routines, and environmental control needs to be documented, acknowledged, and respected, allowing for an individualized sleep routine and respecting person-centered care. However, with high-care demands on limited staff, few environmental options, and shared spaces, oftentimes the approach is more institutionalized. While RAC facilities can offer older adults a safe environment with assistance as required, this may be at the expense of their personal autonomy, privacy, and independence.^30^ The Royal Commission recommends placing people at the center of aged care and this ethos should be extended to the sleep practices of residents. Rather than a “one size fits all” approach, person-centered care would support residents, where possible, in autonomously dictating their own sleep routines and practices and planning and delivering care to support this.

Despite attempts to regulate and limit restrictive practices, levels remain inappropriately high within aged care,^19^ a finding echoed by the very data this study uses.^11^ In fact, recent mandated reporting has identified a current restrictive practices rate at 18% in Australian RAC.^31^ Previous work by our group described the use of physical restraint captured in the same data^14^ and this study provided descriptions of episodes of prolonged periods of environmental and physical restraint for some residents. This results in a multi-pronged negative impact on residents’ sleep. First, physical constraints are not conducive to a sleep-promoting environment for residents. Secondly, prolonged periods of physical inactivity, via environmental restraint, can also have deleterious impacts on residents’ sleep drive. However, it would be remiss not to acknowledge that most residents in RAC settings have high needs for cognitive and behavioral support, and sleep disturbances are common in this population. Presently, there is a dearth of guidance and support for staff working in RAC to understand how best to manage sleep in residents with dementia in a non-pharmacological manner.

In RAC, staff ratios and skill mix can heavily dictate the timing of care provision. The Royal Commission’s final report identified the aged care workforce as being underpaid, understaffed, and undertrained with aged care workers often not having the time or resources to adequately deliver high-quality care.^11^ This research illustrates how this may extend to sleep-related care/practices. Resident sleeplessness is prevalent, with a recent study highlighting the burden felt by nurses as a result of overnight sleep disturbance of residents with over 78% of nurses stating they were regularly confronted by residents with sleep disturbance on night shift.^32^ To mitigate sleeplessness and night wakenings, a holistic approach to optimizing overnight sleep conditions is necessary. These findings are supported by a recent integrative review which found that while RAC nurses understood the implications of sleep disturbances, they were constrained by meeting organizational expectations of provision of overnight care.^33^

As a result of the Royal Commission findings, changes in mandatory minimum care times in RAC were introduced, as well as the requirement that a registered nurse is onsite at all times.^34^ It is not yet known whether these staffing changes implemented in mid-2023 have or will make a difference to the sleep experience of RAC residents described in this research, and further research is warranted to examine the impact and outcomes of these staffing changes. However, a recent study across 25 Australian RAC settings revealed less than 50% adherence to sleep-related evidence-based care indicators.^35^ Given the complexity of the issues raised by institutional care and the challenges in shifting the routine behaviors of staff, it is likely sustainable change will require further investment for practice shift.

Preservation and promotion of autonomy around sleep environment and routines are paramount to empowering capable residents to manage their own sleep. However, environmental measures are also critical to protect sleep in residents. As well as embracing resident-led care, best-practice care should incorporate sufficient staffing, effective risk management policies and practices, and basic sleep-promoting interventions such as reduced overnight lighting and noise, and minimizing overnight care interruptions.

### Limitations

While this study utilized a significant data corpus detailing the experiences of key stakeholders within RAC, the authors acknowledge there are some inherent limitations. The dataset within this study is limited to the submissions within the Royal Commission, so may not be representative of all RAC residents’ experiences, noting that there may be a skew toward those with a negative experience lodging a submission to the commission, resulting in an inherent negative bias within the data set. In addition, given the vast quantity of data, a large dataset methodology was applied with key search terms directly relevant to sleep. Other search terms which have a causal relationship with sleep may have been omitted. Lastly, as the group has already undertaken similar work relating to chemical restraint^14^ within the same dataset, these terms were not included in the current study.^14^

## Conclusion

These findings highlight significant opportunities for improvement in sleep-promoting practices in RAC. Through the lens of person-centered care, we identified how standardized care processes and procedures, while intended to maintain patient safety in terms of pressure injury, continence, and fall risk, often compromise sleep quality and individual autonomy. The tension between delivering standardized safety protocols and supporting person-centered sleep practices represents a key challenge for the sector. Moving forward, balancing person-centered care with safety and quality will require skilled leadership, a strong evidence base, and adequate staffing levels to implement. Success will depend on reimagining how essential care can be delivered in ways that prioritize both individual sleep preferences and necessary clinical oversight.

## Supplemental Material

sj-pdf-1-wjn-10.1177_01939459251324831 – Supplemental material for Sleep in Residential Aged Care: A Secondary Qualitative Analysis of Data from the Australian Royal Commission into Aged Care Quality and Safety Supplemental material, sj-pdf-1-wjn-10.1177_01939459251324831 for Sleep in Residential Aged Care: A Secondary Qualitative Analysis of Data from the Australian Royal Commission into Aged Care Quality and Safety by Aisling Smyth, Patricia Cain, Sabine Pangerl, Christopher Gordon, Kasia Bail and Davina Porock in Western Journal of Nursing Research
