# Supplementary material for: Sleep in Residential Aged Care: A Secondary Qualitative Analysis of Data from the Australian Royal Commission into Aged Care Quality and Safety
Source: West J Nurs Res. 2025 Mar 3;47(6):449–57. doi: 10.1177/01939459251324831 (PMC12069817; doi:10.1177/01939459251324831)
Supplement: sj-pdf-1-wjn-10.1177_01939459251324831 – Supplemental material for Sleep in Residential Aged Care: A Secondary Qualitative Analysis of Data from the Australian Royal Commission into Aged Care Quality and Safety [file sj-pdf-1-wjn-10.1177_01939459251324831.pdf]

The search strategy was developed through review of sleep-related literature in residential aged care and consultation with subject matter experts. Keywords were organised into five main conceptual categories:

1. Primary Sleep Terms:
    - sleep* (including sleeping, sleepers)
    - nap* (including napping)
    - rest* (including resting, restless)
    - insomnia*
2. bedSleep-Related Behaviours:
    - wake* (including waking up, wakening)
    - bedtime
    - evening
    - overnight
    - night* (including nighttime)
    - woken/woken up
    - awake/awoke
    - Sundowning
    - Insomnia* (including insomniac)
3. Care and Assessment Terms:
    - "sleep assessment"
    - "sleep charts"
    - "assess* sleep"
    - "support* sleep"
    - "managing sleep"
    - "light therapy"
4. Sleep Quality Indicators:
    - disturb* (including disturbance, disturbing, disturbed)
    - "poor sleep"
5. Sleep-Related Medications
    - sedat* (including sedatives, sedated)
    - hypnotics
    - temazepam
    - trazodone

    - zolpidem
    - zaleplon
    - barbituates
    - benzodiazepines
    - lorazepam
    - midozolam

Boolean operators (AND, OR) and wildcards (*) were used to ensure comprehensive capture of relevant content. For example:

- sleep* OR nap* OR rest*
- (sleep OR nap) AND (disturb* OR poor)
- "sleep assessment" OR "assess* sleep"
